# Obesity Impacts Mortality and Rate of Revascularizations Among Patients With Acute Myocardial Infarction: An Analysis of the National Inpatient Sample

**DOI:** 10.7759/cureus.11910

**Published:** 2020-12-04

**Authors:** Genaro Velazquez, Trisha Marie A Gomez, Iriagbonse Asemota, Emmanuel Akuna, Pius E Ojemolon, Precious Eseaton

**Affiliations:** 1 Internal Medicine, John H. Stroger, Jr. Hospital of Cook County, Chicago, USA; 2 Anatomical Sciences, St. George's University, St. George's, GRD; 3 Internal Medicine, College of Medicine, University of Benin, Benin City, NGA

**Keywords:** obesity, obesity paradox, acute myocardial infarction, nis, cardiovascular disease

## Abstract

Background

Obesity is now a recognized chronic comorbid condition which is highly prevalent in the United States. Obesity poses several health risks, affecting multiple organ systems. The cardiovascular system is particularly affected by obesity including its role in atherosclerotic disease and hence myocardial infarction (MI) from atheromatous plaque events. However, multiple population-based studies have shown mixed outcomes in obese patients who have acute MI. This study aimed to determine if obesity paradoxically improved outcomes in patients with acute myocardial infarction (AMI) as well as compare outcomes of mild to moderately obese patients and morbidly obese patients to non-obese patients.

Materials and methods

Data was obtained from the Nationwide Inpatient Sample (NIS) for 2016 and 2017. The study included adult patients with a principal discharge diagnosis of AMI. This group was divided into ST segment elevation myocardial infarction (STEMI) and non-ST segment myocardial infarction (NSTEMI). Obese patients were subdivided into two groups: mild-moderate obesity and morbid obesity. Primary outcome compared inpatient mortality. Secondary outcomes included rate of percutaneous coronary intervention (PCI), coronary artery bypass grafting (CABG), composite revascularization, mean length of hospitalization, total hospital charges, and rates of complications.

Results

In patients with STEMI, mild to moderately obese patients had lower odds of mortality (aOR: 0.80, 95% CI: 0.715-0.906, p < 0.001) compared to non-obese patients. However, morbidly obese patients had higher odds of mortality (aOR: 1.26, 95% CI: 1.100-1.446, p < 0.001) compared to non-obese patients. Mild to moderately obese patients had higher odds of composite revascularization (aOR: 1.24, 95% CI: 1.158-1.334, p < 0.001), PCI (aOR: 1.08, 95% CI: 1.054-1.150, p = 0.014), and CABG (aOR: 1.46, 95% CI: 1.313-1.626, p < 0.001).

Conclusion

The degree of obesity affects outcome of patients with AMI. Cardiovascular interventions during hospitalizations for AMI also varied with degree of obesity. This may have affected the outcome, especially among morbidly obese patients.

## Introduction

Obesity is now a recognized chronic comorbid condition which is highly prevalent in the United States. It has a higher prevalence among the middle age female population [1, 2]. Obesity poses several health risks, affecting multiple organ systems [3, 4]. The cardiovascular system is particularly affected by obesity. The mechanism through which obesity affects the cardiovascular system includes adipokine dysregulation, inflammation, increased circulating free fatty acids, increased oxidative stress and adipose tissue hypoxia, ultimately contributing to atherosclerosis and the development of atheromatous plaques [5]. The development and subsequent disruption of atheromatous plaques results in atherothrombosis, which is the hallmark of acute myocardial infarction (AMI) [6]. Studies involving outcomes of AMI in obese population have yielded mixed results [7-11]. Improved outcomes have fueled concepts including metabolically healthy obesity and the obesity paradox relating to cardiovascular diseases. It is also suggested that this paradox may be due to unaccounted confounding factors yet to be objectively identified [12]. This study aimed to determine if obesity paradoxically improved outcomes in patients with AMI as well as compare outcomes of mild to moderately obese patients and morbidly obese patients to non-obese patients.

## Materials and methods

Design and data source

This study was a retrospective cohort study involving adult patients (aged ≥ 18 years) hospitalized for AMI in the US between January 1, 2016 and December 31, 2017. Data was obtained from the Nationwide Inpatient Sample (NIS) database for 2016 and 2017. The NIS is a database of hospital inpatient stays derived from billing data submitted by hospitals to statewide data organizations across the US, covering more than 97% of the US population [13-16]. It approximates a 20% stratified sample of discharges from US community hospitals, excluding rehabilitation and long-term acute care hospitals [17,18]. This dataset is weighted to obtain national estimates [19,20]. Both the 2016 and 2017 database are coded using the International Classification of Diseases, Tenth Revision, Clinical Modification/Procedure Coding System (ICD-10-CM/PCS) [21,22].

Study population

**Figure 1:**
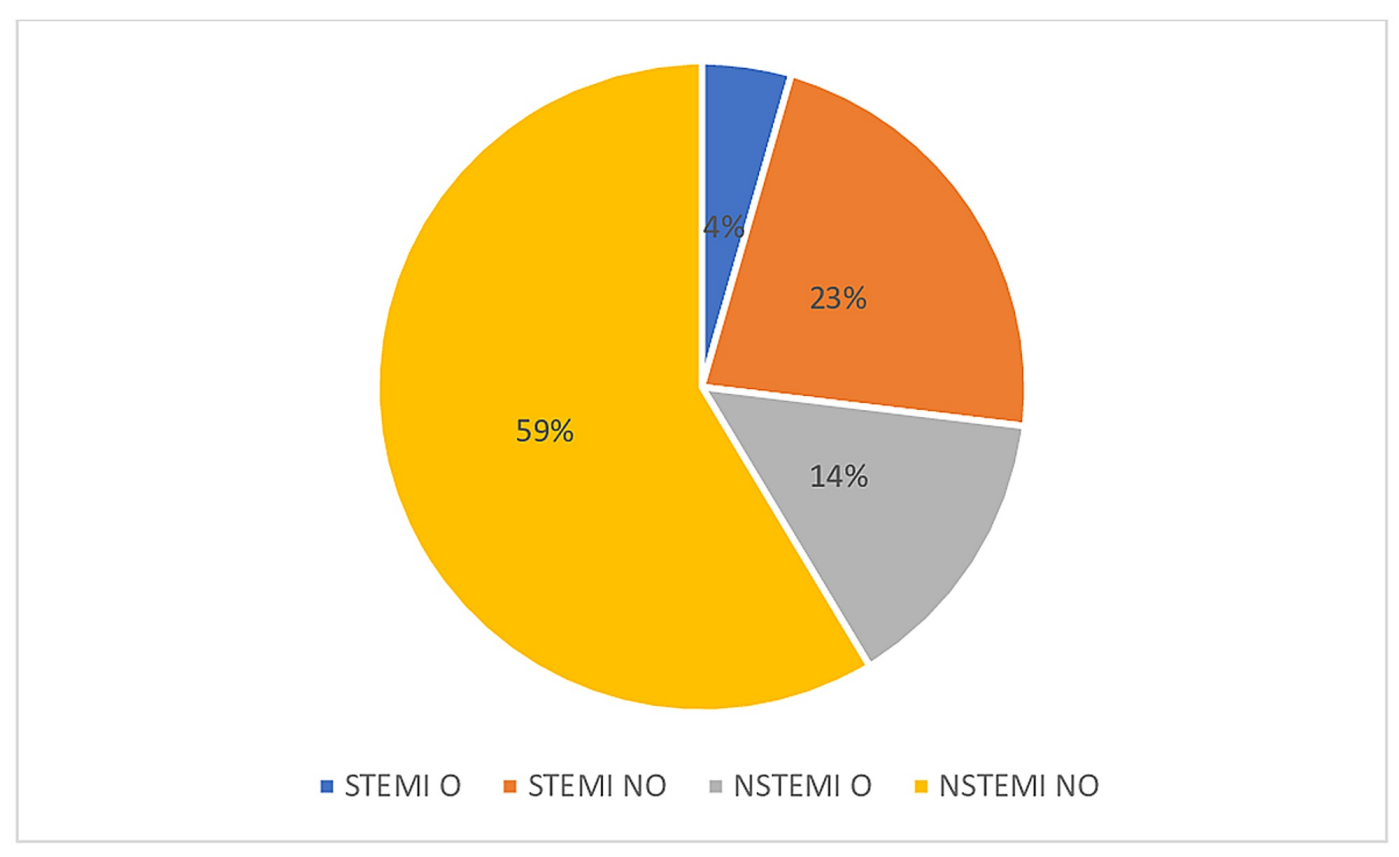
Distribution of Acute Myocardial Infarction Hospitalization O: Obese, NO: Non-obese, STEMI: ST Elevation Myocardial infarction, NTSEMI: Non-ST Elevation Myocardial infarction N/B: % are of total acute myocardial infarction hospitalization

The study included adult patients with a principal discharge diagnosis of AMI (n = 1,299,885). This group was divided into ST segment elevation myocardial infarction (STEMI: I21.0, I21.1, I21.2, I21.3) and non-ST segment myocardial infarction (NSTEMI: I21.4). A total of 349,900 hospitalizations were for STEMI, out of which 16.5%(n = 57,734) were obese and 83.5% (n = 292,166) were non-obese. A total of 949,985 hospitalization were for NSTEMI, out of which 19.8% (n = 188,097) were obese and 80.2% (n = 761,888) were non-obese (Figure 1). Patients were excluded if STEMI or NSTEMI was a secondary diagnosis or if they developed AMI following a procedure. The cohort of patients with AMI was further divided based on the presence of a secondary discharge diagnosis of obesity (E66.0, E66.1, E66.2, E66.8, E66.9, Z68.3, Z68.4). We combined both general codes for obesity as well as BMI specific codes to accurately capture obesity categories. Obese patients were subdivided into two groups: mild-moderate obesity and morbid obesity, using the above codes, correlating with a BMI of 30-39 and 40 and above, respectively. The ICD-10-CM/PCS codes used to obtain the cohort can be found in the Appendix.

Outcome measures

The primary outcome was comparing inpatient mortality among patients with AMI based on presence or absence of obesity. Secondary outcomes in this population included need for percutaneous coronary intervention (PCI) with drug eluting stent and bare metal stent placement, coronary artery bypass grafting (CABG), and composite revascularization (PCI and CABG). Other outcomes included rate of complications including need for electrical cardioversion/defibrillation and odds of having a secondary discharge diagnosis of acute kidney failure (AKI) and cardiogenic shock. We also compared mean length of hospitalization as well as total hospital charges between both groups as measures of healthcare utilization cost.

Statistical analysis

Data was analyzed using Stata® (Statistics and Data) Version 16 software (StataCorp, Texas, USA). All analyses were conducted using the weighted samples for national estimates in adjunct with Healthcare Cost and Utilization Project regulations for using the NIS database. Co-morbidities were calculated as proportions of the cohort and Chi squared test was used to compare association between the non-obese and the obese subgroups. Multivariate regression analysis was done to adjust for possible confounders while calculating the primary and secondary outcomes. The patient and hospital characteristics as well as comorbidities were obtained during literature review. A univariate screen was done to further confirm these factors. Variables with p < 0.2 in univariate screen were included in multivariable regression model. A p-value of 0.05 was set as the threshold for statistical significance in the multivariate regression analysis.

Ethical considerations

The NIS database does not contain patient identifiers. Since 2012, the NIS has also removed state level and hospital identifiers. This has enhanced patient protection and anonymity. This study was exempt from Institutional Review Board approval.

## Results

Characteristics of STEMI patients

Data showed 349,900 hospitalizations were for STEMI during the study period. The prevalence of obesity among patients with STEMI was 16.5%.

Obese patients were significantly younger (59.2 vs 64.3 years, p < 0.001), with a higher proportion of females (34.4 vs 30.2%, p < 0.001). Compared to non-obese patients, obese patients had a higher proportion with comorbidities including hypertension (57.3 vs 50.7%, p < 0.001), diabetes (47.9 vs 28.0%, p < 0.001), smoking history (52.5 vs 51.4%, p = 0.034), congestive heart failure (25.7 vs 23.0%, p < 0.001) and chronic kidney disease (10.5 vs 8.5%, p < 0.001) (Table 1).

**Table 1:** Patient and hospital characteristics of STEMI and NSTEMI by obesity #: For 2017; *: Secondary diagnosis CABG: Coronary artery bypass grafting, CHF: Congestive heart failure, CKD: Chronic kidney disease, COPD: Chronic obstructive pulmonary disease, CVA: Cerebrovascular accident, IHD: Ischemic heart disease, NSTEMI: Non-ST segment elevation myocardial infarction, PCI: Percutaneous coronary intervention, STEMI: ST segment elevation myocardial infarction.

Variable		STEMI N = 349,900			NSTEMI N = 949,985	
	Obese, %	Non-obese, %	p-value	Obese, %	Non-obese, %	p-value
Patient characteristics						
Percent	16.5	83.5		19.8	80.2	
Mean Age, years	59.2	64.3	<0.001	63.0	69.5	<0.001
Females	34.4	30.2	<0.001	42.8	40.1	<0.001
Racial distribution			<0.001			<0.001
White	73.1	72.0		70.2	70.5	
Black	9.4	8.2		13.4	11.4	
Hispanic	8.2	7.8		8.6	8.1	
Others	9.3	12.0		7.8	10.0	
Insurance type			<0.001			<0.001
Medicaid	37.4	48.5		54.2	66.3	
Medicare	12.8	10.6		11.3	8.7	
Private	42.6	34.1		30.0	21.4	
Uninsured	7.2	6.8		4.5	3.6	
Charlson Comorbidity Index score			<0.001			<0.001
1	25.7	34.7		20.5	21.6	
2	32.4	30.8		22.5	20.5	
≥3	41.9	34.5		57.0	55.9	
Median annual income expected for patient’s zip code, US$^#^			<0.001			<0.001
1-43,999	28.6	28.5		32.7	31.3	
44,000-55,999	28.2	27.5		28.1	27.4	
56,000-73,999	25.0	23.9		23.4	23.0	
≥74,000	18.2	20.1		15.8	18.3	
Comorbidities*						
Hypertension	57.3	50.7	<0.001	47.6	45.8	<0.001
Diabetes	47.9	28.0	<0.001	60.2	39.1	<0.001
Smoking history	52.5	51.4	0.034	49.2	47.7	<0.001
Atrial fibrillation/flutter	14.1	14.4	0.372	20.6	22.1	<0.001
CHF	25.7	23.0	<0.001	39.6	37.2	<0.001
CKD	10.5	8.5	<0.001	23.8	21.6	<0.001
Dialysis dependence	1.2	1.1	0.643	3.5	4.0	<0.001
Dyslipidemia	72.4	61.3	<0.001	75.3	65.6	<0.001
Chronic IHD	87.1	84.2	<0.001	83.1	80.1	<0.001
Old PCI	1.4	1.5	0.874	1.7	1.8	0.148
Old CABG	4.1	4.5	0.102	10.6	12.9	<0.001
Pacemaker	0.8	1.2	<0.001	2.5	4.0	<0.001
Prior CVA	1.1	1.3	0.112	2.3	2.7	<0.001
Liver disease	4.8	4.3	0.012	3.6	3.0	<0.001
COPD	12.2	11.7	0.103	21.6	20.2	<0.001
Supplemental O_2_	1.3	0.9	<0.001	3.7	2.8	<0.001
Hypothyroidism	9.0	8.4	0.045	13.3	13.1	0.171
Electrolyte disorders	20.6	20.1	0.186	22.1	22.2	0.837
Anemia	15.9	14.8	0.004	26.2	25.7	0.040
Hospital characteristics						
Hospital region			<0.001			<0.001
Northeast	15.8	17.1		16.0	18.6	
Midwest	25.8	22.2		25.2	21.7	
South	39.8	40.6		40.8	41.0	
West	18.6	20.1		18.0	18.7	
Hospital bed size			0.462			0.030
Small	15.1	14.5		16.6	17.4	
Medium	29.6	29.8		30.5	30.8	
Large	55.3	55.7		52.9	51.8	
Urban location	94.3	93.3	<0.001	93.0	91.6	<0.001
Teaching hospital	68.8	67.6	0.035	68.0	65.6	<0.001

Primary outcome in STEMI patients: in-hospital mortality

The in-hospital mortality for patients with STEMI was 8.0% overall. Mild to moderately obese patients had lower odds of mortality (aOR: 0.80, 95% CI: 0.715-0.906, p < 0.001) compared to non-obese patients. However, morbidly obese patients had higher odds of mortality (aOR: 1.26, 95% CI: 1.100-1.446, p < 0.001) compared to non-obese patients (Tables 2, 3).

**Table 2:** Clinical outcomes of STEMI in mild to moderately obese patients *: Statistically significant, AKI: Acute kidney failure, aOR: adjusted odds ratio, BMS: Bare metal stent, CABG: Coronary artery bypass grafting, CI: Confidence interval, DES: Drug eluting stent, PCI: Percutaneous coronary intervention, STEMI: ST segment elevation myocardial infarction.

Outcome	Mild-moderate, %	Nonobese, %	aOR (95% CI)	p-value
Primary outcome				
In-hospital mortality	5.1	8.4	0.80 (0.715 – 0.906)	<0.001*
Secondary outcomes				
Mean Length of stay, days (95% CI)	4.1 (3.9 - 4.2)	4.0 (4.0 - 4.1)	0.00 (-0.107 – 0.102)	0.965
Mean total hospital charges, US$ (95% CI)	113000 (109300 – 116600)	107600 (105400 – 109800)	2200 (-800 – 5100)	0.151
PCI with DES	69.9	65.1	1.12 (1.054 – 1.180)	<0.001*
PCI with BMS	8.6	9.2	0.93 (0.851 – 1.023)	0.138
PCI	77.4	73.5	1.08 (1.016 – 1.150)	0.014*
CABG	7.6	4.7	1.46 (1.313 – 1.626)	<0.001*
Revascularization	83.9	77.7	1.24 (1.158 – 1.334)	<0.001*
AKI	15.6	15.7	1.10 (1.013 – 1.193)	0.023*
Electrical cardioversion/defibrillation	4.6	4.0	1.18 (1.041 – 1.326)	0.009*
Cardiogenic shock	11.5	13.3	0.91 (0.837 – 0.992)	0.032*

**Table 3:** Clinical outcomes of STEMI in morbidly obese patients *: Statistically significant, AKI: Acute kidney failure, aOR: adjusted odds ratio, BMS: Bare metal stent, CABG: Coronary artery bypass grafting, CI: Confidence interval, DES: Drug eluting stent, PCI: Percutaneous coronary intervention, STEMI: ST segment elevation myocardial infarction.

Outcome	Morbid obesity, %	Nonobese, %	aOR (95% CI)	p-value
Primary outcome				
In-hospital mortality	7.8	8.4	1.26 (1.100 – 1.446)	0.001*
Secondary outcomes				
Mean Length of stay, days (95% CI)	4.7 (4.5 – 4.9)	4.0 (4.0 – 4.1)	0.34 (0.170 – 0.516)	<0.001*
Mean total hospital charges, US$ (95% CI)	120500 (115900 – 125200)	107600 (105400 – 109800)	7900 (3900 – 12000)	<0.001*
PCI with DES	63.5	65.1	0.91 (0.841 – 0.973)	0.007*
PCI with BMS	10.0	9.2	1.11 (0.994 – 1.235)	0.063
PCI	72.5	73.5	0.92 (0.846 – 0.991)	0.030*
CABG	6.8	4.7	1.35 (1.170 – 1.559)	<0.001*
Revascularization	78.8	77.7	0.99 (0.911 – 1.086)	0.905
AKI	21.0	15.7	1.47 (1.333 – 1.610)	<0.001*
Electrical cardioversion/defibrillation	5.2	4.0	1.31 (1.128 – 1.526)	<0.001*
Cardiogenic shock	13.6	13.3	1.05 (0.944 – 1.160)	0.383

Secondary outcomes in STEMI patients

Mild to moderately obese patients had higher odds of composite revascularization (aOR: 1.24, 95% CI: 1.158-1.334, p < 0.001), PCI (aOR: 1.08, 95% CI: 1.054-1.150, p = 0.014), and CABG (aOR: 1.46, 95% CI: 1.313-1.626, p < 0.001). These patients had lower odds of cardiogenic shock (aOR: 0.91, 95% CI: 0.837-0.992, p = 0.032), but higher odd of AKI (aOR: 1.10, 95% CI: 1.013-1.193, p = 0.023) and electrical cardioversion or defibrillation (aOR: 1.18, 95% CI: 1.041-1.326, p = 0.009) when compared to non-obese patients (Table 2).

There was no difference in rate of composite revascularization between morbidly obese and non-obese patients (aOR: 0.99, 95% CI: 0.911-1.086, p = 0.905). Morbidly obese patients had lower odds of PCI (aOR: 0.92, 95% CI: 0.846-0.991, p = 0.030), but higher odds of CABG (aOR: 1.35, 95% CI: 1.170-1.559, p < 0.001) and AKI (aOR: 1.47, 95% CI: 1.333-1.610, p < 0.001) compared to non-obese patient (Table 3).

Characteristics of NSTEMI patients

Obese patients admitted for NSTEMI had a significantly lower mean age compared to non-obese patients (63.0 vs 69.5 years, p < 0.001). Obese patients had higher proportion of medical comorbidities (Table 1).

Primary outcome in NSTEMI patients: in-hospital mortality

A total of 949,985 hospitalizations involved patients with NSTEMI. The in-hospital mortality for patients with NSTEMI was 3.5% overall. Mild to moderately obese patients with NSTEMI had a lower adjusted odds ratio for mortality (aOR: 0.73, 95% CI: 0.660-0.811, p < 0.001) when compared to non-obese patients with NSTEMI. Patients with morbid obesity had no difference in mortality (aOR: 0.95, 95% CI: 0.854-1.055, p = 0.333) compared to non-obese patients (Tables 4, 5).

**Table 4:** Clinical outcomes of NSTEMI in mild to moderately obese patients *: Statistically significant, AKI: Acute kidney failure, aOR: adjusted odds ratio, BMS: Bare metal stent, CABG: Coronary artery bypass grafting, CI: Confidence interval, DES: Drug eluting stent, PCI: Percutaneous coronary intervention, NSTEMI: Non-ST segment elevation myocardial infarction.

Outcome	Mild-moderate, %	Nonobese, %	aOR (95% CI)	p-value
Primary outcome				
In-hospital mortality	2.0	3.8	0.73 (0.660 – 0.811)	<0.001*
Secondary outcomes				
Mean Length of stay, days (95% CI)	4.8 (4.7 – 4.9)	4.5 (4.4 – 4.5)	0.28 (0.205 – 0.351)	<0.001*
Mean total hospital charges, US$ (95% CI)	96400 (94100 – 38800)	83800 (82200 – 85400)	7100 (5400 – 8700)	<0.001*
PCI with DES	36.4	31.2	1.06 (1.028 – 1.100)	<0.001*
PCI with BMS	3.2	3.2	0.97 (0.887 – 1.051)	0.420
PCI	39.3	34.1	1.05 (1.017 – 1.088)	0.003*
Revascularization	54.1	42.3	1.31 (1.262 – 1.350)	<0.001*
CABG	15.0	8.3	1.65 (1.576 – 1.747)	<0.001*
AKI	19.6	20.0	1.06 (1.014 – 1.108)	0.011*
Electrical cardioversion/defibrillation	1.4	1.2	1.12 (0.985 – 1.284)	0.081
Cardiogenic shock	3.2	3.6	0.93 (0.855 – 1.016)	0.110

**Table 5:** Clinical outcomes of NSTEMI in morbidly obese patients *: Statistically significant, AKI: Acute kidney failure, aOR: adjusted odds ratio, BMS: Bare metal stent, CABG: Coronary artery bypass grafting, CI: Confidence interval, DES: Drug eluting stent, PCI: Percutaneous coronary intervention, NSTEMI: Non-ST segment elevation myocardial infarction.

Outcome	Morbid obesity, %	Nonobese, %	aOR (95% CI)	p-value
Primary outcome				
In-hospital mortality	2.6	3.8	0.95 (0.854 – 1.055)	0.333
Secondary outcomes				
Mean Length of stay, days (95% CI)	5.4 (5.3 – 5.5)	4.5 (4.4 – 4.5)	0.63 (0.536 – 0.723)	<0.001*
Mean total hospital charges, US$ (95% CI)	100000 (97200 – 102900)	83800 (82200 – 85400)	10000 (7800 – 12100)	<0.001*
PCI with DES	32.7	31.2	1.02 (0.983 – 1.064)	0.269
PCI with BMS	2.7	3.2	0.85 (0.759 – 0.941)	0.002*
PCI	35.1	34.1	1.00 (0.960 – 1.039)	0.942
Revascularization	46.9	42.3	1.11 (1.071 – 1.156)	<0.001*
CABG	12.0	8.3	1.35 (1.268 – 1.437)	<0.001*
AKI	24.9	20.0	1.32 (1.264 – 1.386)	<0.001*
Electrical cardioversion/defibrillation	1.6	1.2	1.28 (1.111 – 1.465)	0.001
Cardiogenic shock	3.4	3.6	0.87 (0.791 – 0.962)	0.006

Secondary outcomes in NSTEMI patients

Mild to moderately obese patients with NSTEMI had significantly higher adjusted odds ratio of composite revascularization (aOR: 1.31, 95% CI: 1.262-1.350, p < 0.001), PCI (aOR: 1.05, 95% CI: 1.017-1.088, p = 0.003), and CABG (aOR: 1.65, 95% CI: 1.576-1.747, p < 0.001). They also had longer length of hospitalization and higher total hospital charges compared to non-obese patients (Table 4).

Morbidly obese patients with NSTEMI had significantly higher adjusted odds ratio of composite revascularization (aOR: 1.11, 95% CI: 1.071-1.156, p < 0.001) and CABG (aOR: 1.35, 95% CI: 1.268-1.437, p < 0.001). They also had longer length of hospitalization and higher total hospital charges compared to non-obese patients (Table 5).

## Discussion

Obesity is prevalent in patients with AMI. Although more males had AMI, obesity was associated with a higher prevalence in females with AMI. This is congruent with the overall higher prevalence of obesity in females in the US [3]. Obese patients were significantly younger on hospitalization in both the STEMI and NSTEMI groups, likely due to the association of obesity with early development of coronary disease [23, 24]. Whites, Blacks and Hispanics with AMI also had a higher proportion of obese patients suggesting that the racial disparity in obese population also reflects in these patients [3].

Among patients with STEMI, mild to moderately obese patients had lower odds of inpatient mortality. However, morbidly obese patients had higher odds of inpatient mortality compared to nonobese patients. This showed that the severity of obesity likely impacted mortality in STEMI patients. A conclusion reached by Das et al. showed that morbid obesity was independently associated with worse outcomes among patients with STEMI [9]. This finding is at variance with a study by Dhoot et al., which demonstrated lower odds of mortality in morbidly obese patients [25]. This study did not stratify AMI which could have been a confounding factor.

In NSTEMI, mild to moderately obese patients had a significantly lower adjusted odds for inpatient mortality compared to nonobese patients. There was no difference in mortality between morbidly obese patients and non-obese patients with NSTEMI.

The study demonstrated higher odds of composite revascularization in mild to moderately obese patients with STEMI. There was no difference in composite revascularization between morbidly obese patients and non-obese patients. This finding may be associated with better outcomes in patients with mild-moderate compared to morbidly obese patients who had STEMI. Among patients with NSTEMI, both mild-moderate and morbidly obese patients had higher odds of revascularization compared to non-obese patients.

There was increasingly higher hospital resource utilization with levels of obesity. This is seen in the rising length of hospitalization and the total hospital charges. This is similar to a study by Champagne-Langabeer et al., which showed morbidly obese patients had longer treatment times compared to other patients with MI [26]. This places significant stress on the healthcare institutions amid limited resources.

Various reasons have been postulated for the better outcome in mild to moderately obese patients including lower incidence of undernutrition, weight loss, possible presence of protective cytokine, greater metabolic reserves and possibly differing obesity phenotypes [11, 24]. We also suggest that although obesity is a risk factor for cardiovascular diseases, in patients with AMI, the earlier age of presentation as well as the statistically significant higher rates of interventions including PCI and CABG, procedures with known mortality benefits, help to offset this risk. However, the poorer outcomes in morbidly obese patients suggest that with progression of obesity, there is a proportional increase in cardiovascular risk.

Our study has some important limitations. NIS database is subject to non-randomization. The NIS is an administrative database that uses ICD-10 codes to characterize diagnoses and hospitalization events [27]. BMI could not be coded on a linear scale, as BMI ranges rather than individual BMI values are available. The disability associated with obesity could not be measured using the NIS database. Data in NIS is on hospitalizations, rather than individual patients. Hence if the same patient gets admitted on more than one occasion, that patient will be counted multiple time [28]. There is no reliable way to determine if secondary diagnoses preceded or started in the index hospitalization. NIS studies cannot establish causation, but only association.

## Conclusions

The degree of obesity affects outcome of patients with AMI. Cardiovascular interventions during hospitalizations for AMI also varied with degree of obesity. This may have affected the outcome, especially among morbidly obese patients. The reasons for these differences are not clear. However, morbidly obese patients had poorer outcomes compared to patients with only mild-moderate obesity. Increased revascularization procedures may improve outcomes in obese patients. Further studies are required to elucidate factors responsible for this paradox as well as identifying the point at which these variables no longer improve outcomes in obese people.

## Appendices

**Table 6:** Used ICD-10 codes ACS: Acute Coronary Syndrome, STEMI: ST Elevation Myocardial infarction, NSTEMI: Non-ST Elevation Myocardial Infarction, UA: Unstable Angina, RA: Rheumatoid Arthritis, MI: Myocardial Infarction, PCI: Percutaneous Coronary Intervention, CABG: Coronary Artery Bypass Graft, PTCA: Percutaneous Transluminal Coronary Angioplasty, PCI DES: Percutaneous Coronary Intervention with Drug Eluting Stent, PCI BMS: Percutaneous Coronary Intervention with Bare Metal Stent, IABP: Intra-aortic Balloon Pump, PEAD: Percutaneous External Assist Devices.

	ICD-10 codes
Diagnosis codes	
STEMI	I21.0, I21.1, I21.2, I21.3
NSTEMI	121.4
Obesity	E66.0, E66.1, E66.2, E66.8, E66.9, Z68.3, Z68.4
Procedure codes	
PTCA	02703ZZ, 02704ZZ, 02713ZZ, 02714ZZ, 02723ZZ, 02724ZZ, 02733ZZ, 02734ZZ
PCI BMS	02703D6, 02703DZ, 02704D6, 02704DZ, 02703E6, 02703EZ, 02704E6, 02704EZ, 02703F6, 02703FZ, 02704F6, 02704FZ, 02703G6, 02703GZ, 02704G6, 02704GZ, 02713D6, 02713DZ, 02714D6, 02714DZ, 02713E6, 02713EZ, 02714E6, 02714EZ, 02713F6, 02713FZ, 02714F6, 02714FZ, 02713G6, 02713GZ, 02714G6, 02714GZ, 02723D6, 02723DZ, 02724D6, 02724DZ, 02723E6, 02723EZ, 02724E6, 02724E6, 02724EZ, 02723F6, 02723FZ, 02724F6, 02724FZ, 02723G6, 02723GZ, 02724G6, 02724GZ, 02733D6, 02733DZ, 02734D6, 02734DZ, 02733E6, 02733EZ, 02734E6, 02734EZ, 02733F6, 02733FZ, 02733FZ, 02734F6, 02733G6, 02733GZ, 02734G6, 02734GZ
PCI DES	0270346, 027034Z, 0270446, 027044Z, 0270356, 027035Z, 0270456, 027045Z, 0270366, 027036Z, 0270466, 027046Z, 0270376, 027037Z, 0270476, 027047Z, 0271346, 027134Z, 0271446, 027144Z, 0271356, 027135Z, 0271456, 027145Z, 0271366, 027136Z, 0270376, 0271466, 027146Z, 0271376, 027137Z, 0271476, 027147Z, 0272346, 027234Z, 0272446, 027244Z, 0272356, 027235Z, 0272456, 027245Z, 0272366, 027236Z, 027246Z, 0272376, 027237Z, 0272476, 027035Z, 027247Z, 0273346, 027334Z, 0273446, 027344Z, 0273356, 027335Z, 0273456, 027345Z, 0273366, 027336Z, 0273466, 027346Z, 0273376, 027337Z, 027045Z, 0273476, 027347Z
IABP	5A02210, 5A02110
PEAD	02HA0RJ, 02HA3RJ, 02HA4RJ, 5A02116, 5A0211D, 5A02216, 5A0221D, 02HA3RZ, 5A02216
Intracoronary artery thrombolytic infusion	3E07017, 3E07317
CABG	0210093, 0210098, 0210099, 021009C, 021009F, 021009W, 02100A3, 02100A8, 02100A9, 02100AC, 02100AF, 02100AW, 0211093, 0211098, 0211099, 021109C, 021109F, 021109W, 02110A3, 02110A8, 02110A9, 02110AC, 02110AF, 02110A, 0212093, 0212098, 0212099, 021209C, 021209F, 021209W, 02120A3, 02120A8, 02120A9, 02120AC, 02120AF, 02120AW, 0213093, 0213098, 0213099, 021309C, 021309F, 021309W, 02130A3, 02130A8, 02130A9, 02130AC, 02130AF, 02130AW
Comorbidities	
Dyslipidemia	E78
Old MI	I252
Old PCI	Z9861
Old CABG	Z951
Old pacemaker	Z950
Atrial fibrillation/flutter	I48
Chronic obstructive pulmonary disease	J41, J42, J43, J44
Old stroke	I69
Hypertension	I10
Peripheral vascular disease	I739
Hypothyroidism	E03
Diabetes Mellitus Type 1&2	E10, E11
Congestive heart Failure	I50
Chronic Kidney Disease	N18
Liver disease	K70, K71, K72, K73, K74, K75, K76, K77
Electrolyte derangement	E870, E871, E872, E873, E874, E875, E876
Oxygen dependence	Z9981
Smoking	Z87891, F17200
Anemia	D50, D51, D52, D53, D55, D56, D57, D58, D59, D60, D61, D62, D63, D64
